# ChromBERT-tools: a versatile toolkit for context-specific regulatory representations of transcription regulators across different cell types

**DOI:** 10.1093/bioinformatics/btag423

**Published:** 2026-06-19

**Authors:** Qianqian Chen, Zhanhao Li, Zhaowei Yu, Yong Zhang

**Affiliations:** State Key Laboratory of Cardiovascular Diseases and Medical Innovation Center, Institute for Regenerative Medicine, Department of Neurosurgery, Shanghai East Hospital, Shanghai Key Laboratory of Signaling and Disease Research, Frontier Science Center for Stem Cell Research, School of Life Sciences and Technology, Tongji University, Shanghai 200092, China; State Key Laboratory of Cardiovascular Diseases and Medical Innovation Center, Institute for Regenerative Medicine, Department of Neurosurgery, Shanghai East Hospital, Shanghai Key Laboratory of Signaling and Disease Research, Frontier Science Center for Stem Cell Research, School of Life Sciences and Technology, Tongji University, Shanghai 200092, China; State Key Laboratory of Cardiovascular Diseases and Medical Innovation Center, Institute for Regenerative Medicine, Department of Neurosurgery, Shanghai East Hospital, Shanghai Key Laboratory of Signaling and Disease Research, Frontier Science Center for Stem Cell Research, School of Life Sciences and Technology, Tongji University, Shanghai 200092, China; State Key Laboratory of Cardiovascular Diseases and Medical Innovation Center, Institute for Regenerative Medicine, Department of Neurosurgery, Shanghai East Hospital, Shanghai Key Laboratory of Signaling and Disease Research, Frontier Science Center for Stem Cell Research, School of Life Sciences and Technology, Tongji University, Shanghai 200092, China

## Abstract

**Summary:**

Representations that encode the genome-wide regulatory behavior of transcription regulators provide a foundation for flexible transcription modeling and in silico regulatory analysis. Existing regulator representations are commonly derived from gene co-expression, motif annotations, or static protein features, which capture useful but limited aspects of regulator identity but do not directly model how regulators participate in region-specific regulatory programs across the genome. ChromBERT addresses this gap by learning context-aware regulatory representations from large-scale ChIP-seq data. However, routine bioinformatics applications require lightweight, accessible, and modular tools for generating, adapting, and interpreting these representations in user-defined biological contexts. Here, we present ChromBERT-tools, a user-oriented toolkit built upon ChromBERT that converts its regulatory representation framework into practical workflows for customizable analysis across cellular contexts. ChromBERT-tools provides command-line interfaces and Python APIs organized into three functional layers: representation generation, predictive modeling, and regulatory interpretation. The representation generation layer produces representations of genomic regions and transcription regulators. The predictive modeling layer fine-tunes ChromBERT for genome-wide regulatory activity prediction through classification or regression tasks, with optimized implementation to reduce running time and computational resource requirements. The regulatory interpretation layer supports inference of the context-specific roles of *cis*-regulatory elements and transcription regulators. These modules can be used independently or integrated into end-to-end workflows, enabling flexible analyses across diverse datasets. ChromBERT-tools lowers the barrier to applying context-specific regulatory representations in routine genomic analyses.

**Availability and Implementation:**

ChromBERT-tools is freely available at https://github.com/TongjiZhanglab/ChromBERT-tools, with documentation at https://chrombert-tools.readthedocs.io/en/latest/. A frozen archival snapshot is available on Zenodo under DOI: 10.5281/zenodo.20094206.

## 1 Introduction

Transcription regulators (TRs) serve as master regulators of cellular identity, orchestrating dynamic regulatory landscapes through precise, combinatorial interactions at *cis*-regulatory elements (CREs) (Kim and Wysocka 2023, Stampfel *et al.* 2015). Recent transformer-based models have successfully captured distinct dimensions of these regulators: single-cell foundation models, such as scGPT, excel at mapping cell-type-specific gene-gene interaction networks from expression data (Cui *et al.* 2024), while protein language models, such as ESM-2, effectively encode intrinsic biochemical and structural potentials from amino acid sequences (Lin *et al.* 2023). Nevertheless, these approaches remain disconnected from direct genome-wide binding landscapes. By relying on static sequences or co-expression correlations, they fail to explicitly model the *trans*-acting mechanisms of TFs, the direct genome-wide binding landscapes and combinatorial co-occupancy patterns required to understand cooperative regulation. To bridge this gap, we pre-trained ChromBERT on large-scale TR ChIP-seq data, demonstrating that context-specific regulatory representations can effectively capture these missing physical mechanisms and generalize across cell types (Yu *et al.* 2026). Unlike sequence- or expression-derived baselines, these representations explicitly encode context-specific TR–chromatin associations and combinatorial TR–TR co-occupancy, thereby helping to bridge this disconnect between computational models and the dynamic genome context. In practice, however, applying ChromBERT-derived representations to new biological questions requires more than access to the pre-trained model. Downstream regulatory analyses often involve heterogeneous datasets, cell types, and analytical objectives, including representation extraction, task-specific prediction, and biological interpretation. These diverse use cases are difficult to support through a single predefined workflow, limiting the accessibility of context-specific regulatory representations for routine bioinformatics analyses.

To address this need, we developed ChromBERT-tools, a lightweight and user-oriented software toolkit built on top of ChromBERT for regulatory analysis on user-defined data. ChromBERT-tools provides modular command-line interfaces and Python APIs for generating regulatory representations of genomic regions and transcription regulators, fine-tuning ChromBERT for genome-wide regulatory activity prediction, and interpreting context-specific regulatory mechanisms involving *cis*-regulatory elements and transcription regulators. These modules can be used independently or integrated into end-to-end workflows, allowing users to adapt ChromBERT-derived representations to diverse datasets and biological questions. Together, these capabilities extend ChromBERT from a pre-trained regulatory representation model into a practical bioinformatics platform.

## 2 Toolkit overview

**Figure 1:**
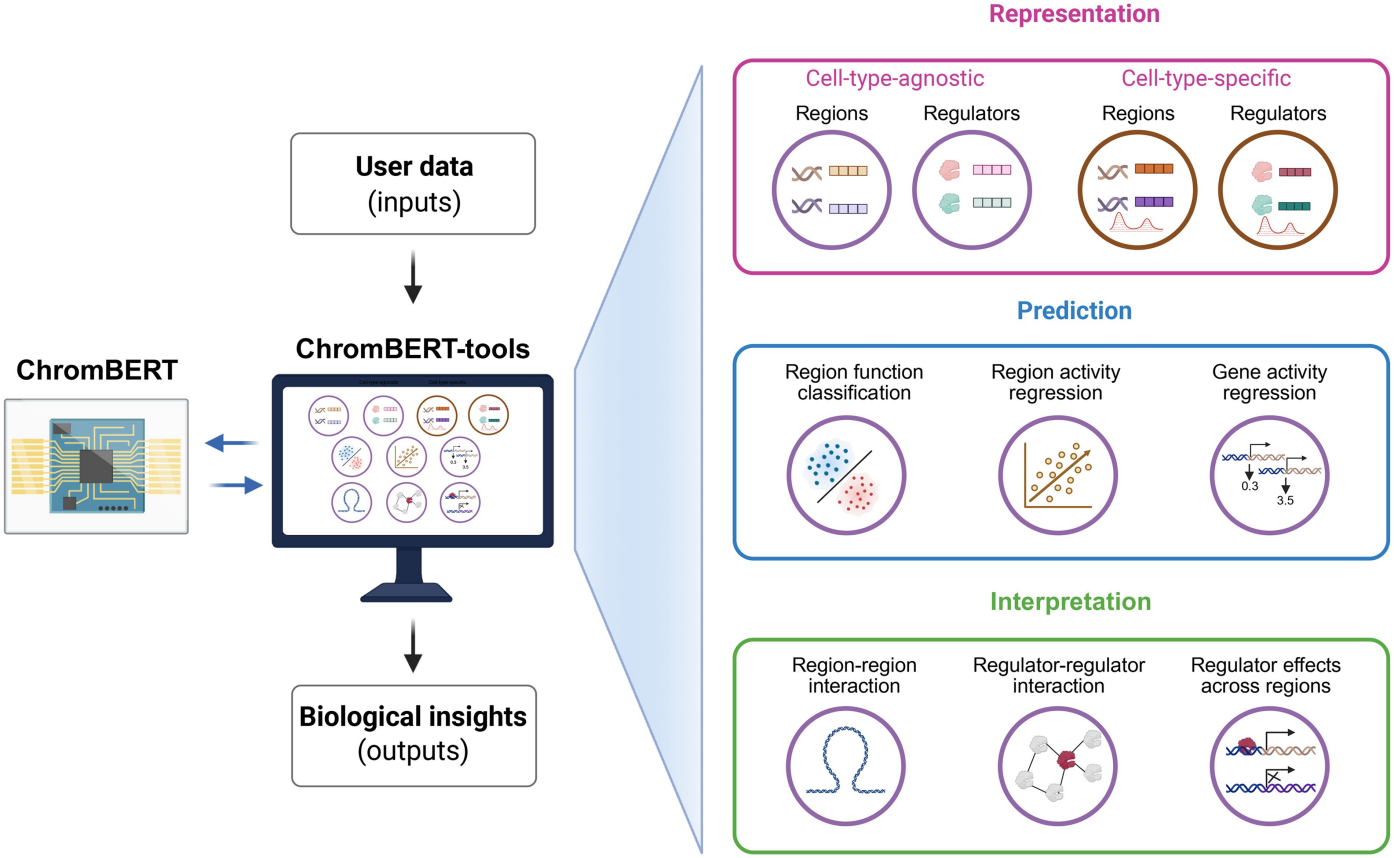
Overview of ChromBERT-tools. ChromBERT-tools is a modular software framework built on the ChromBERT foundation model to support practical regulatory analysis on user-defined data. It converts context-specific regulatory representations into biological insights through three functional layers: representation generation, predictive modeling, and regulatory interpretation. The representation generation layer supports cell-type-agnostic and cell-type-specific modes for generating region and regulator representations. The predictive modeling layer includes modules for region function classification, region activity regression, and gene activity regression. The regulatory interpretation layer enables region–region interaction analysis, regulator–regulator interaction analysis, and analysis of regulator effects between region groups. Created in BioRender. Chen, Q. (2026) https://BioRender.com/lcibnuw.

ChromBERT-tools is organized into three functional layers: representation generation, predictive modeling, and regulatory interpretation (Fig. 1). This modular design allows users to apply individual modules to focused tasks, such as directly extracting cell-type-agnostic region or regulator representations from the pre-trained model, or to combine multiple modules into end-to-end workflows for more comprehensive analyses. For example, users can fine-tune ChromBERT-derived representations to predict cell-type-specific regulatory activity and subsequently interpret the cell-type-specific roles of *cis*-regulatory elements and regulators within the fine-tuned representation space. All modules are accessible through both Python APIs and command-line interfaces, supporting flexible use in exploratory analyses and streamlined pipeline execution.

To facilitate practical use on user data, ChromBERT-tools supports human and mouse reference assemblies across multiple genomic resolutions, ranging from 200 bp to 4 kb for human and including 1 kb for mouse. Most modules require a limited set of standardized inputs, typically species, resolution, regions of interest, regulator names, and optional cell-type-specific files. The toolkit further automates key practical steps, including input processing, model loading, fine-tuning, prediction, and downstream interpretation. Together, these features reduce the need for custom pipeline development and make regulatory analyses substantially easier to deploy in practice.

### 2.1 Context-specific regulatory representation generation

Context-specific regulatory representation generation is a core capability of ChromBERT-tools and provides the foundation for downstream analyses. This layer supports two complementary modes. In the cell-type-agnostic mode, representations are extracted directly from the pre-trained ChromBERT model, enabling general characterization of genomic regions and transcription regulators. In the cell-type-specific mode, representations are generated from models fine-tuned to predict genome-wide regulatory activity, such as chromatin accessibility, in user-defined cellular contexts. This allows the representation space to be adapted to the biological context of interest (Fig. 1). Details of the fine-tuning procedure are described in Section 2.2, with additional implementation information provided in the Supplementary Materials, available as supplementary data at *Bioinformatics* online.

### 2.2 Predictive modeling

Region representations learned by ChromBERT encode properties of individual regulators and their combinatorial interactions, providing regulatory information beyond embeddings derived solely from DNA sequence. Accordingly, the second functional layer of ChromBERT-tools provides Python APIs and command-line interfaces for predictive modeling based on these representations. ChromBERT-tools supports three task types: region function classification, which can be formulated as either multiclass or binary classification; region activity regression, which predicts region-level activities such as cell-type-specific accessibility or accessibility fold change between two cell states; and gene activity regression, which predicts gene expression levels or expression fold changes between two cell states using TSS-centered representations that incorporate the promoter region together with upstream and downstream flanking regions. Additional details are provided in the Supplementary Materials, available as supplementary data at *Bioinformatics* online.

Because these predictive tasks involve fine-tuning on user-provided datasets, ChromBERT-tools provides two training modes to balance computational efficiency and predictive accuracy: Fast mode is intended for rapid iteration and down-samples the training input to at most 20 000 regions, substantially reducing running time while maintaining performance comparable to full training (Fig. S1, available as supplementary data at *Bioinformatics* online). Full mode uses all available training regions and is intended for settings in which maximizing predictive accuracy is preferred.

### 2.3 Context-specific regulatory interpretation

The regulatory representations generated by ChromBERT-tools provide a basis for interpreting regulatory relationships across regions, regulators, and cellular contexts. Because these representations are embedded in a shared high-dimensional space, their similarities and differences can be used to compare regulatory functions and infer context-specific regulatory relationships. Accordingly, the third functional layer of ChromBERT-tools provides Python APIs and command-line interfaces for representation-based regulatory interpretation.

ChromBERT-tools supports three interpretation modes. First, region–region interaction analysis uses cosine similarity between region representations to identify genomic regions with similar regulatory properties and candidate functional relationships, such as putative enhancer–promoter links. Second, regulator–regulator interaction analysis uses cosine similarity between regulator representations to identify transcription regulators with similar or potentially cooperative regulatory roles. Third, regulator difference analysis between region groups compares the representation of the same regulator across two groups of regions with distinct regulatory activities. Larger representation differences highlight regulators whose representation changes more strongly between groups, providing a simple way to prioritize candidates potentially associated with group-specific regulatory differences. Additional implementation details are provided in the Supplementary Materials, available as supplementary data at *Bioinformatics* online.

### 2.4 Implementation

ChromBERT-tools is implemented in Python (v3.9+) and distributed with a containerized environment (Apptainer/Singularity) to simplify installation and ensure consistent deployment across systems. It can also be installed and deployed directly from source. The source code and documentation, including command-line options, input/output specifications, and detailed tutorials, are publicly available (https://github.com/TongjiZhanglab/ChromBERT-tools).

To assess practical usability, we evaluated the computational cost of ChromBERT-tools across representative tasks from different functional layers using batch sizes of 1, 4, 8, 16, 32, and 64 and input region set sizes of 5 k, 10 k, 20 k, and 50 k (Fig. S2, available as supplementary data at *Bioinformatics* online). Resource usage depended primarily on batch size, task type, and input size. At practical batch sizes of 4–8, GPU peak memory ranged from approximately 2–10 GB across tasks, whereas CPU peak memory ranged from approximately 1–3 GB, suggesting compatibility with a broad range of commonly available devices. Running time further depended on the number of input regions. For 20 k input regions, fine-tuning tasks required less than 20 minutes per epoch, whereas forward-only tasks completed within 8 minutes.

## 3 Applications

We highlight a representative end-to-end workflow in ChromBERT-tools, built by integrating core modules across the representation generation, predictive modeling, and interpretation layers.

In this workflow, *embed_region* first generates cell-type-agnostic representations for genomic regions of interest. These representations are then used by *region_activity_regression*, which fine-tunes the model with cell-type-specific chromatin accessibility peaks and signals to obtain context-specific representations. The fine-tuned model is subsequently applied by *interpret_region_region_interactions* to generate cell-type-specific representations for promoter regions and distal regions of interest and quantifies their representation similarity. In promoter-centered analyses, distal regions with higher similarity to a given promoter can be prioritized as candidate enhancers linked to that promoter in the corresponding cell type, thereby enabling cell-type-specific enhancer–promoter link identification.

We illustrate this application at the *ZNF879* locus by comparing HFF and hESC cells. ChromBERT-tools identified distinct putative enhancers and candidate enhancer–promoter links in the two cell types, and these links were supported by Micro-C chromatin interaction data (Fig. S3, available as supplementary data at *Bioinformatics* online). Notably, several distal regions near *ZNF879* were accessible in both HFF and hESC cells, suggesting that chromatin accessibility alone was insufficient to resolve their cell-type-specific regulatory roles. After cell-type-specific fine-tuning, however, the representation similarity between each distal region and the *ZNF879* promoter became context-dependent. In each cell type, the distal region with the highest similarity to the *ZNF879* promoter corresponded to the Micro-C-supported interaction in that same cell type, whereas distal regions that contacted the promoter only in the other cell type showed lower similarity. Together, these results indicate that fine-tuned representations can distinguish shared accessible regions from cell-type-specific enhancer-promoter links. This example demonstrates how ChromBERT-tools can support context-specific regulatory interpretation by integrating representation learning, predictive modeling, and representation-based interaction analysis.

## 4 Discussion

ChromBERT-tools is a lightweight, user-oriented, and efficient toolkit for generating, adapting, and interpreting regulatory representations of genomic regions and transcription regulators across cellular contexts. Built on the ChromBERT foundation model, it converts context-specific regulatory representations learned from large-scale ChIP-seq data into practical workflows for user-defined genomic analysis. ChromBERT-tools is intended to complement, rather than replace, existing regulatory analysis strategies. Motif scanning combined with chromatin accessibility remains a useful and interpretable approach for predicting TF binding and co-binding, particularly for sequence-specific transcription factors with well-characterized motifs. Recent sequence-based genome foundation models also provide powerful frameworks for sequence-centered regulatory prediction (Avsec *et al.* 2026). In contrast, ChromBERT-tools focuses on making ChIP-seq-informed regulatory representations practically accessible for user-defined analyses. Because these representations are learned from genome-wide regulator co-occupancy patterns, they can support representation-based interpretation of context-specific regulatory relationships, including analyses involving cofactors or chromatin-associated regulators that are not readily captured by motif information alone.

A major practical contribution of ChromBERT-tools is that it lowers the engineering barrier for applying ChromBERT-derived representations to new biological questions. The toolkit organizes representation generation, predictive modeling, and regulatory interpretation into reusable command-line interfaces and Python APIs, allowing users to apply individual modules to focused tasks or combine multiple modules into end-to-end workflows. It also automates key steps including input processing, model loading, fine-tuning, prediction, evaluation, and downstream interpretation. Flexible training modes allow users to balance computational efficiency and predictive performance, supporting both rapid exploratory analyses and more comprehensive full-data modeling. Together with containerized deployment, documentation, tutorials, and computational benchmarks, these features help users estimate resource requirements and apply ChromBERT-tools to their own datasets without developing customized pipelines around the original ChromBERT model.

A current limitation of ChromBERT-tools is that it presently supports only human and mouse, reflecting the scope of the available pre-trained ChromBERT models. Because ChromBERT learns regulatory representations from structured TF-binding profiles derived from large-scale, species-specific ChIP-seq data rather than from DNA sequence, direct extension to additional species would require comparable species-specific ChIP-seq resources and corresponding pre-trained models. Such resources are currently unavailable at sufficient scale for most organisms. Future work could address this limitation by pre-training ChromBERT on additional species as suitable ChIP-seq resources become available, or by aligning DNA sequence foundation model embeddings with the ChromBERT representation space through transfer learning or contrastive learning, providing a possible route toward cross-species generalization.

## Supplementary Material

btag423_Supplementary_Data

## Data Availability

The datasets used in this study are available from the Hugging Face repository TongjiZhanglab/chrombert at https://huggingface.co/datasets/TongjiZhanglab/chrombert.
