# Supplementary material for: ChromBERT-tools: a versatile toolkit for context-specific regulatory representations of transcription regulators across different cell types
Source: Bioinformatics. 2026 Jun 19;42(6):btag423. doi: 10.1093/bioinformatics/btag423 (PMC13313317; doi:10.1093/bioinformatics/btag423)
Supplement: btag423_Supplementary_Data [file btag423_supplementary_data.pdf]

Supplementary Material for

ChromBERT-tools: A versatile toolkit for context-specific regulatory representations of transcription regulators across different cell types

Qianqian Chen[1], Zhanhao Li[1], Zhaowei Yu[1,*], Yong Zhang[1,*]

[1]State Key Laboratory of Cardiovascular Diseases and Medical Innovation Center, Institute for Regenerative Medicine, Department of Neurosurgery, Shanghai East Hospital, Shanghai Key Laboratory of Signaling and Disease Research, Frontier Science Center for Stem Cell Research, School of Life Sciences and Technology, Tongji University, Shanghai, 200092, China

*Correspondence: zhaoweiyu@tongji.edu.cn, yzhang@tongji.edu.cn

This PDF file includes

Supplementary methods
Supplementary figures
Supplementary references

Supplementary methods

Data processing

ChromBERT-tools accepts several types of user inputs depending on the analysis module, including genomic region files, gene identifiers, regulator names, and optional cell-type-specific signal tracks.

Genomic regions. Users provide genomic regions as a BED file. ChromBERT-tools uses the first three columns of the BED file, corresponding to chromosome, start, and end coordinates. The regions are sorted by genomic coordinates and mapped to ChromBERT reference bins using *bedtools*[1] *intersect -a <ref_bins> -b <user_regions> -f 0.5 -F 0.5 -wa -wb -e*. For each matched interval, ChromBERT-tools outputs the original user-provided region coordinates and the matched reference-bin identifier, recorded in the build_region_index column. One user region can be mapped to multiple ChromBERT bins when applicable.

Genes. For gene-centered analyses, users can provide gene symbols or Ensembl gene IDs. ChromBERT-tools uses a precomputed gene-to-bin mapping table, in which each gene is assigned to the ChromBERT reference bin containing its transcription start site (TSS), defined from the GRCh38 Ensembl GTF (release 110). Given a gene symbol or Ensembl gene ID, the toolkit retrieves the corresponding TSS-associated reference-bin identifier.

Regulators. For regulator-level analyses, users provide regulator names or cell-type-specific regulator names as cell-type–factor pairs. ChromBERT-tools resolves these inputs using curated regulator lists and metadata tables. The current release includes 1,073 human regulators and 701 mouse regulators, together with metadata for available cell-type–factor pairs, which are used for input validation and lookup.

Cell-type-specific signal tracks. For modules requiring chromatin accessibility or cell-type-specific signal information, users provide ATAC-seq or DNase-seq signal tracks in BigWig format. ChromBERT-tools summarizes signal values from these BigWig files over mapped intervals derived from user-provided regions using pybbi.stackup[2] using the mean signal value over each interval by default. Optionally, summarized values can be normalized by the global mean signal of the BigWig track.

Input processing in region activity regression for cell-type-specific modeling. ChromBERT-tools constructs fine-tuning inputs from user-provided raw chromatin accessibility data (peak BED and BigWig tracks). Using the region-to-bin mapping and BigWig signal summarization steps, ChromBERT-tools computes the mean accessibility signal $s_{c,b}$for each reference bin b in the target cell type c. As a baseline, ChromBERT-tools provides a precomputed mean accessibility signal $s_{baseline,b}$for each reference bin,

aggregated across all available accessibility profiles in the metadata. ChromBERT-tools applies a log transform to both signals and defines the supervised training target (fold-change) as:

$$y_b = log_2(1 + s_{c,b}) - log_2(1 + s_{baseline,b})$$

ChromBERT-tools defines highly accessible regions as reference bins with y_b > 1 and selects the top 1,000 bins ranked by y_b, and ChromBERT-tools defines background regions by first filtering bins with $s_{c,b}$ > 0 and $s_{baseline,b}$ > 0 and then selecting the 1,000 bins with the smallest $|y_b|$.

Input processing in region activity regression for accessibility-based transition analysis. ChromBERT-tools constructs fine-tuning inputs from user-provided chromatin accessibility data of the start and end cell states (peak BED and BigWig tracks). ChromBERT-tools defines training regions as the union of peaks from both states together with all protein-coding gene TSS bins. For each reference bin b, ChromBERT-tools computes the mean accessibility signal in the start and end states, denoted $s_{start,b}$ and $s_{end,b}$.The supervision target is the log-transformed accessibility change:

$$y_b = log_2(1 + s_{end,b}) - log_2(1 + s_{start,b})$$

Input processing in gene activity regression for transcriptome-based transition analysis. For transcriptome-based transition analysis, ChromBERT-tools constructs fine-tuning inputs from user-provided RNA expression profiles and defines a gene-level supervision target for each protein-coding gene:

$$y_g = ln(1 + e_{end,g}) - ln(1 + e_{start,g})$$

where $e_{*,g}$ denotes the expression level of gene g (e.g., TPM).

ChromBERT-tools defines gained-accessibility regions as reference bins with y_b > 1 and selects the top 1,000 bins ranked by y_b. Unchanged bins are defined by requiring $s_{end,b}$ > 0 and $s_{start,b}$ > 0, and then selecting the 1,000 bins with the smallest $|y_b|$. Similarly, up-regulated genes are defined as genes with y_g > 1, and unchanged genes are selected as the 1,000 genes with the smallest $|y_g|$ (optionally restricting to $|y_g|$ < 0.5 to exclude large expression changes).

Input processing in region function classification for region-class discrimination. ChromBERT-tools constructs fine-tuning inputs from two or more user-defined region sets. Each region set is assigned a class label, such as A, B, or additional user-defined classes, forming a supervised region classification task that supports both binary and multiclass settings.

All fine-tuning datasets are split into training/validation/test sets with an 8:1:1 ratio.

Model fine-tuning

For classification tasks, ChromBERT-tools uses binary cross-entropy (BCE) loss and reports validation AUPRC for binary classification, whereas it uses cross-entropy loss and

reports validation macro-averaged F1 score for multiclass classification.

For regression tasks, ChromBERT-tools uses root mean squared error (RMSE) loss and reports the Pearson correlation coefficient (PCC) between predictions and targets on the validation set. ChromBERT-tools applies early stopping when the monitored validation metric does not improve for 5 consecutive validation checks (min_delta = 0.01), except for expression prediction, where early stopping is applied after 10 consecutive validation checks without improvement. ChromBERT-tools selects and saves the checkpoint with the best validation performance (highest AUPRC/PCC, depending on the task). Training runs for up to 10 epochs. Gradients are accumulated over 64 mini-batches. Validation is performed five times per epoch (every 20% of an epoch). By default, ChromBERT-tools freezes the first six Transformer blocks and fine-tunes only the final two blocks and the task-specific head.

ChromBERT-tools provides two fine-tuning modes to balance computational efficiency and predictive accuracy. In Fast mode, ChromBERT-tools subsamples up to 20,000 training regions for fine-tuning, whereas Full mode uses all available training regions after preprocessing.

Model validation

ChromBERT-tools evaluates the fine-tuned model on a held-out test set, reporting PCC for regression tasks, AUPRC for binary classification tasks, and macro-averaged F1 score for multiclass classification tasks. To mitigate occasional training instability caused by random initialization and local optima, ChromBERT-tools automatically restarts fine-tuning with a new initialization if the primary test metric falls below 0.2; this process is attempted up to three times. ChromBERT-tools also reports additional test-set metrics, including Spearman correlation coefficient, MSE, and MAE for regression; AUROC, MCC, F1 score, precision, and recall for binary classification; and accuracy, macro-averaged precision, macro-averaged recall, macro-averaged F1 score, and MCC for multiclass classification.

Representations generation

Regulator and region representations were generated using the *embed_regulator* and *embed_region* subcommands, respectively. For regulator representation generation, users provide target regulator names (--regulator) and a BED file defining the focus regions (--region). For region representation generation, users provide the focus-region BED file (--region). The input format follows the data-processing procedure described above.
By default, both subcommands extract regulatory embeddings from the pretrained ChromBERT model. When a cell-type-specific fine-tuned checkpoint is provided (--ft-ckpt), embeddings are generated from the supplied checkpoint to obtain cell-type-specific region or regulator representations. Alternatively, when cell-type-specific accessibility signal tracks and peak files are provided (--cell-type-bw and --cell-type-peak) without an existing checkpoint, ChromBERT-tools first fine-tunes ChromBERT to the specified cellular context and then generates

embeddings from the resulting fine-tuned model. Additional parameters, including --genome and --resolution, specify the target genome and genomic resolution for representation generation. By default, ChromBERT-tools uses the human genome at 1-kb resolution.

Predictive modeling

The region function classification model was implemented as the *region_function_classification* subcommand. Users provide functionally distinct sets of genomic regions as BED files (--function-bed). The subcommand fine-tunes ChromBERT using these region sets as user-defined functional classes, thereby adapting the model to the corresponding regulatory contexts. Input processing and model fine-tuning procedures follow those described above.

The region activity regression model was implemented as the *region_activity_regression* subcommand. This subcommand fine-tunes ChromBERT using region-level activity profiles from a user-defined cellular context as regression targets, thereby adapting the model to that context. For cell-type-specific modeling, users provide accessible regions (--acc-peak1) and accessibility signals (--acc-signal1) from the target cell type. ChromBERT-tools then fine-tunes ChromBERT to predict cell-type-specific chromatin accessibility signals. For cell-state transition modeling, users provide accessibility peaks (--acc-peak1 and --acc-peak2) and signal tracks (--acc-signal1 and --acc-signal2) from the starting and target states. ChromBERT-tools then fine-tunes ChromBERT to predict chromatin accessibility fold changes between the two states. Input processing and model fine-tuning procedures follow those described above.

The gene activity regression model was implemented as the *gene_activity_regression* subcommand. This subcommand fine-tunes ChromBERT using gene-level activity profiles from a user-defined cellular context as regression targets, thereby adapting the model to that context. For cell-type-specific modeling, users provide a gene expression file from the target cell type (--exp-tpm1). ChromBERT-tools then fine-tunes ChromBERT to predict cell-type-specific gene expression levels. For cell-state transition modeling, users provide gene expression files from the starting and target states (--exp-tpm1 and --exp-tpm2). ChromBERT-tools then fine-tunes ChromBERT to predict gene expression fold changes between the two states. For gene activity prediction, ChromBERT-tools uses representations of TSS-centered genomic regions as input features. By default, the model incorporates four upstream and four downstream neighboring bins around the TSS-associated region to capture local regulatory information. Input processing and model fine-tuning procedures follow those described above.

Interpretation analysis

ChromBERT-tools computes embedding similarity using cosine similarity and defines embedding difference as 1 − cosine similarity.

Region–region interaction analysis

Region–region interaction analysis was implemented in the *interpret_region_region_ interactions* subcommand. This module quantifies the embedding-based similarity between genomic regions using cosine similarity and supports two input modes. For either mode, users may provide a cell-type-specific fine-tuned model checkpoint to perform cell-type-specific analysis. If no checkpoint is provided, the pretrained ChromBERT model is used by default.

In the single-region-set mode for enhancer–promoter interaction analysis, embeddings of user-provided regions (--region) are compared with embeddings of TSS-associated region bins. Gene annotations are provided as built-in metadata for 55,240 human genes and 42,112 mouse genes, including TSS coordinates and their corresponding ChromBERT region bins. Promoter regions are defined as TSS-associated region bins in this analysis. Because the analysis is performed at the gene-annotation level, multiple genes whose TSSs fall within the same ChromBERT bin are retained as separate genes and can form separate region-gene pairs with the same promoter bin. Users may restrict the analysis to specific genes by providing gene names or gene IDs (--gene or --gene-id). If no genes are specified, all annotated genes are considered, and region-gene pairs are generated between each gene TSS and input regions located within a default ±250 kb window. This window can be adjusted using the --*distance_min* and --*distance_max* parameters. Cosine similarity is then calculated for each region–gene pair.

In the two-region-set mode, pairwise cosine similarities are computed between regions from two input files (--region; --region2) within the specified distance interval, enabling region-set-to-region-set comparison.

Regulator–regulator interaction analysis

Regulator–regulator interaction analysis was implemented in the *interpret_regulator_ regulator_interactions* subcommand. This module quantifies embedding-based similarity between regulators using cosine similarity. Users may optionally provide a cell-type-specific fine-tuned model checkpoint for cell-type-specific analysis; otherwise, the pretrained ChromBERT model is used by default.

Given regulators of interest and user-defined context regions (--region; --regulator), ChromBERT-tools generates region-specific regulator representations, averages the representations of each regulator across the provided regions, and computes pairwise cosine similarities between regulators. An edge is defined between two regulators if their cosine similarity exceeds a user-defined threshold (--quantile). By default, this threshold is set to the 98th percentile of all pairwise regulator-regulator cosine similarities, and the resulting edges are interpreted as putative regulator interactions. For subnetwork visualization, ChromBERT-tools extracts the k-hop neighborhood (--k-hop) of a user-specified regulator in the regulator–regulator interaction graph, with k = 1 used by default.

Regulator difference analysis between region groups

Regulator difference analysis between region groups was implemented in the *interpret_regulator_effects_between_region_groups* subcommand. This module quantifies regulator embedding differences between region groups as 1 − cosine similarity. Given user-provided region groups (--region1-file; --region2-file), ChromBERT-tools generates region-specific regulator embeddings, averages the embeddings of each regulator within each group, and then calculates the embedding difference between groups. Larger embedding differences highlight regulators whose representations change more strongly across region groups. Users may optionally provide a cell-type-specific fine-tuned model checkpoint for cell-type-specific analysis; otherwise, the pretrained ChromBERT model is used by default.

Application

Identification of driver regulators during cell state transition

ChromBERT-tools identifies candidate driver regulators during cell-state transitions by combining the *region_activity_regression* or *gene_activity_regression* subcommand in the predictive modeling layer with the *interpret_regulator_effects_between_region_groups* subcommand in the regulatory interpretation layer. For chromatin accessibility-based analysis, ChromBERT is fine-tuned using accessibility changes between the starting and target states. For transcriptome-based analysis, ChromBERT is fine-tuned using gene expression changes between the two states. The resulting transition-fine-tuned model is then used to generate transition-specific regulator embeddings, which are compared between gained-accessibility regions and unchanged background regions in the accessibility-based workflow, or between up-regulated genes and unchanged background genes in the transcriptome-based workflow. Regulators with stronger transition-associated embedding differences are prioritized as candidate driver regulators.

This workflow is implemented as the end-to-end *predict_transition_driver_regulators* subcommand. For the accessibility-based workflow, the minimum required inputs are accessibility peaks and signal tracks from the two states (--acc-peak1, --acc-peak2, --acc-signal1, and --acc-signal2). For the transcriptome-based workflow, the minimum required inputs are expression profiles from the two states (--exp-tpm1 and --exp-tpm2). The output is a ranked list of candidate transition driver regulators.

Predicting TF-binding regions

ChromBERT-tools predicts TF-binding profiles in cell types without matched ChIP-seq data using the prompt-enhanced fine-tuned model released with ChromBERT. ChromBERT-tools integrates region and regulator embeddings generated from pretrained ChromBERT with cell-type-specific prompt embeddings, and uses the fine-tuned model to predict regulator occupancy across user-provided candidate regulatory regions. In the current release, ChromBERT-tools implements the DNase-based workflow, in which the cell-type prompt is a DNase-seq embedding generated by ChromBERT. The RNA-seq-based prompt setting explored in the original ChromBERT study is not currently supported as an end-to-end workflow in ChromBERT-tools. Regulator selection is currently limited to the

regulator vocabulary supported by the released ChromBERT resources, including 1,073 regulators in the Cistrome-Human-6K regulator set. Therefore, ChromBERT-tools does not currently support arbitrary new regulators that were not included in the original ChromBERT regulator vocabulary.

This workflow is implemented as the end-to-end *predict_tf_binding_regions* subcommand. The minimum required inputs are target cell type–factor pairs (--cistrome) and target regions (--region). The output is the predicted TF-binding probability for each input region.

Supplementary figures

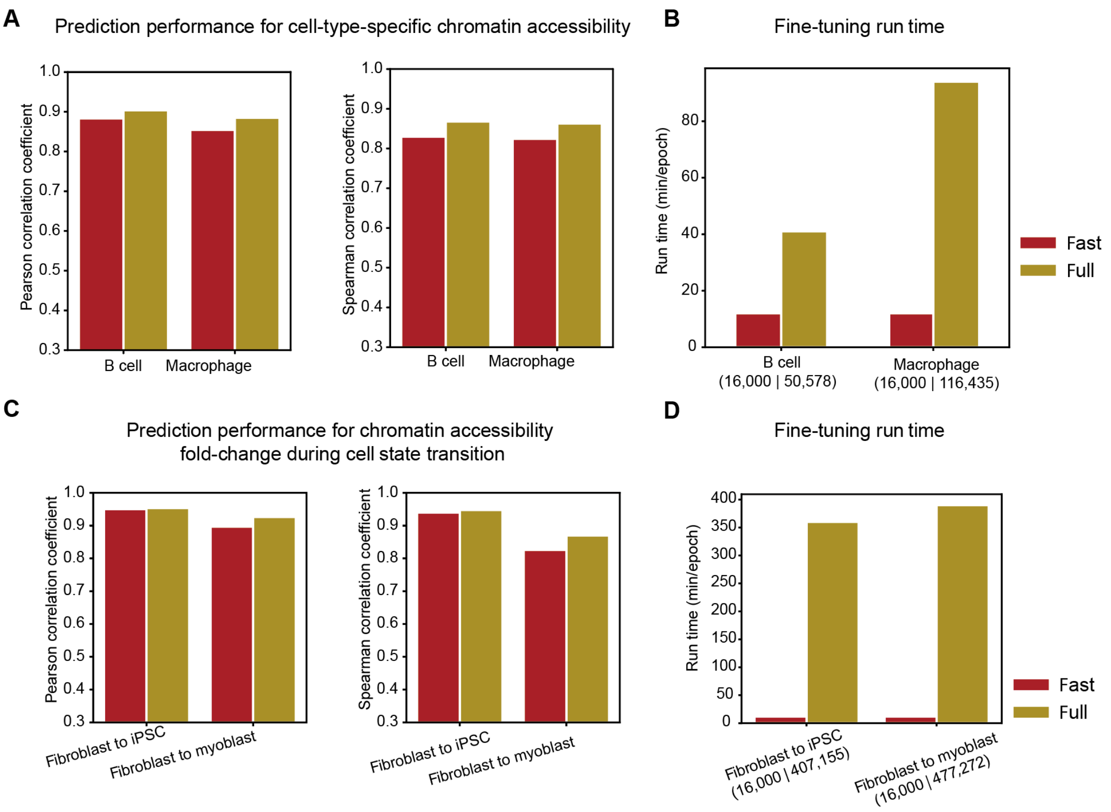

Figure S1. Benchmark of prediction performance and run time in Fast and Full modes.

Benchmarks were performed on an NVIDIA A100 40GB GPU using the default parameters in ChromBERT-tools for two representative predictive modeling tasks.

(A) Prediction performance in Fast and Full modes for cell-type-specific fine-tuning. ChromBERT was fine-tuned to predict genome-wide accessibility signals for a specific cell type. See Supplementary Methods for details. DNase-seq datasets: B cell (ENCSR468AKF[3]) and macrophage (ENCFF593WXK[3]).

(B) Fine-tuning run time per epoch for the task in (A). Early stopping occurred at epoch 3. X-axis values are shown as Fast|Full format and indicate the number of training regions used in each mode.

(C) Prediction performance in Fast and Full modes for transition-specific fine-tuning. ChromBERT was fine-tuned to predict accessibility fold-change between two cell states (target versus source cell type). See Supplementary Methods for details. DNase-seq datasets: fibroblast (ENCFF184KAM[4]), iPSC (ENCFF540VPT[4]), and myoblast (ENCFF647RNC[4]).

(D) Fine-tuning run time per epoch for the task in (C). Training stopped early at epoch 3 for Fast mode and epoch 5 for Full mode. X-axis values are shown as Fast|Full format and indicate the number of training regions used in each mode.

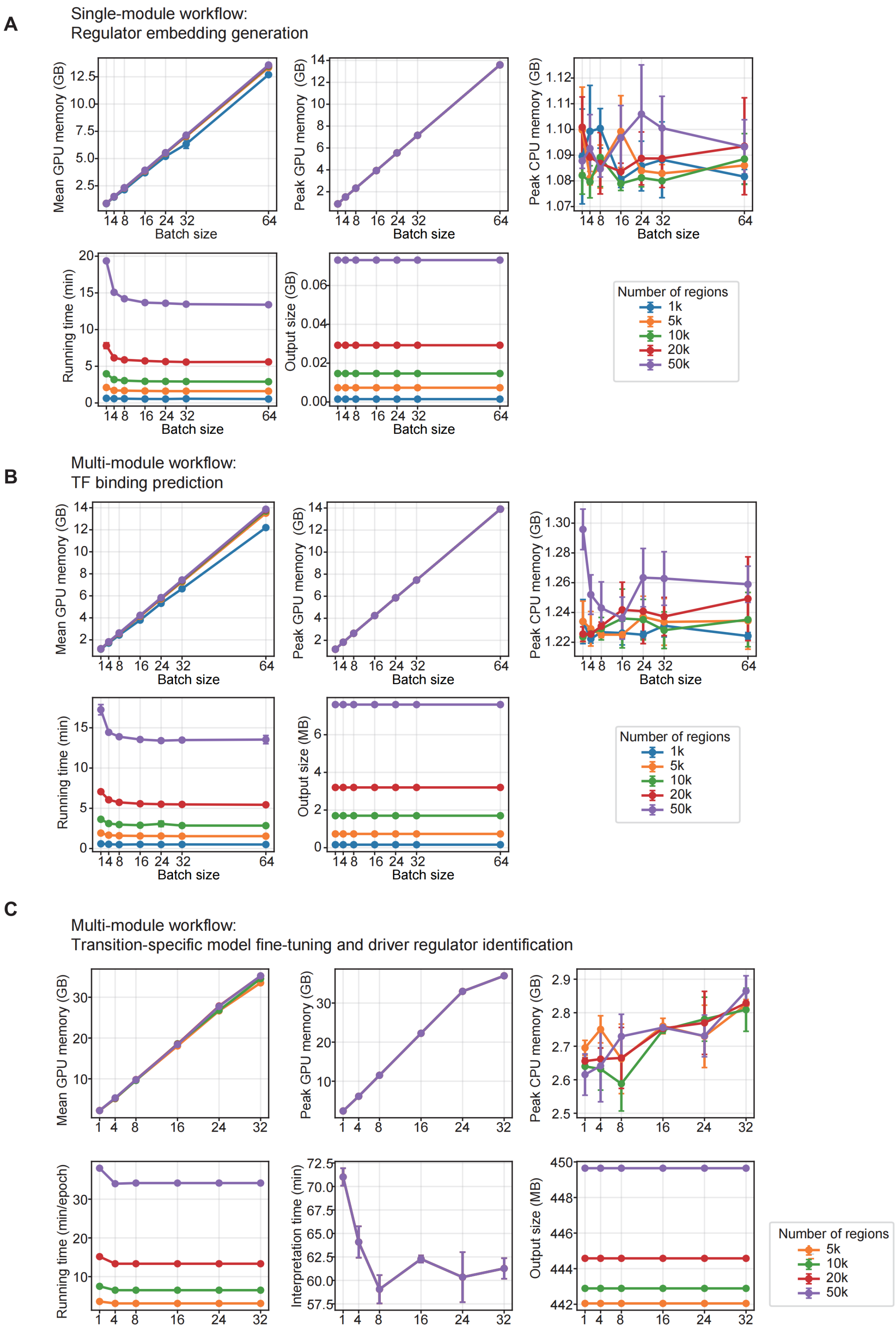
A
Single-module workflow:
Regulator embedding generation
Mean GPU memory (GB)
Peak GPU memory (GB)
Peak CPU memory (GB)
Running time (min)
Output size (GB)
Batch size
Number of regions
1k
5k
10k
20k
50k
B
Multi-module workflow:
TF binding prediction
Mean GPU memory (GB)
Peak GPU memory (GB)
Peak CPU memory (GB)
Running time (min)
Output size (MB)
Batch size
Number of regions
1k
5k
10k
20k
50k
C
Multi-module workflow:
Transition-specific model fine-tuning and driver regulator identification
Mean GPU memory (GB)
Peak GPU memory (GB)
Peak CPU memory (GB)
Running time (min/epoch)
Interpretation time (min)
Output size (MB)
Number of regions
5k
10k
20k
50k

Figure S2. Computational resource usage of representative ChromBERT-tools tasks. Computational cost was evaluated on an NVIDIA A100 40GB GPU across different batch sizes and numbers of input regions. Metrics include mean GPU memory, peak GPU memory, peak CPU memory, running time, and output storage. (A) Resource usage for regulator embedding generation. For a given batch size and number of input regions, this panel shows resource usage for single regulator embedding generation. (B) Resource usage for TF binding prediction using the representation generation and predictive modeling modules. (C) Resource usage for identifying transition driver regulators from DNase-seq data using the representation generation, predictive modeling, and interpretation modules. Fine-tuning and interpretation running times were measured separately.

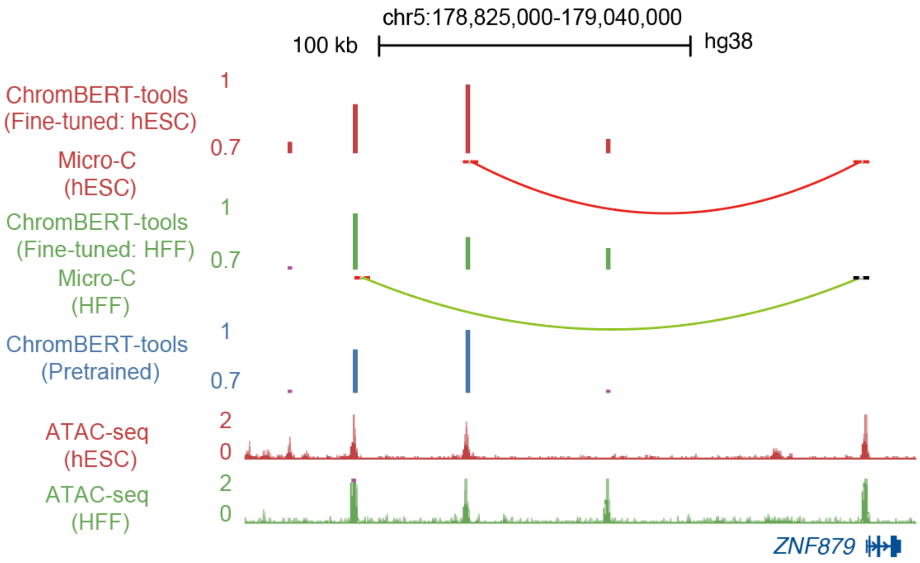

Figure S3. Cell-type-specific interpretation of enhancer–promoter loops.

UCSC Genome Browser views showing representative enhancer–promoter contacts at the *ZNF879* locus (chr5:178,825,000–179,040,000) and their embedding similarities. Only loops linking accessible distal regions to the target promoter are shown, with displayed distal regions restricted to 50–250 kb from the target promoter. Tracks are shown from top to bottom: hESC fine-tuned embedding similarity, hESC Micro-C(4DNES21D8SP8[5]), HFF fine-tuned embedding similarity, HFF Micro-C (4DNESWST3UBH[5]), pretrained embedding similarity, hESC ATAC-seq (GSE109524[6]), and HFF ATAC-seq (GSE79609[6]). The cell-type-specific model was fine-tuned for 3 epochs in Fast mode with a batch size of 4 on an NVIDIA A100 40 GB GPU, reaching a peak GPU memory usage of 8 GB and requiring 30 – 40 min.

Supplementary references

1. Quinlan, A.R. & Hall, I.M. BEDTools: a flexible suite of utilities for comparing genomic features. *Bioinformatics* **26**, 841-2 (2010).
2. Kent, W.J., Zweig, A.S., Barber, G., Hinrichs, A.S. & Karolchik, D. BigWig and BigBed: enabling browsing of large distributed datasets. *Bioinformatics* **26**, 2204-2207 (2010).
3. Consortium, E.P. An integrated encyclopedia of DNA elements in the human genome. *Nature* **489**, 57-74 (2012).
4. Thurman, R.E. *et al.* The accessible chromatin landscape of the human genome. *Nature* **489**, 75-82 (2012).
5. Krietenstein, N. *et al.* Ultrastructural Details of Mammalian Chromosome Architecture. *Mol Cell* **78**, 554-565 e7 (2020).
6. Li, Q.V. *et al.* Genome-scale screens identify JNK-JUN signaling as a barrier for pluripotency exit and endoderm differentiation. *Nat Genet* **51**, 999-1010 (2019).
